# The Astrophysical Formation of Asymmetric Molecules and the Emergence of a Chiral Bias †

**DOI:** 10.3390/life9010029

**Published:** 2019-03-16

**Authors:** Adrien D. Garcia, Cornelia Meinert, Haruna Sugahara, Nykola C. Jones, Søren V. Hoffmann, Uwe J. Meierhenrich

**Affiliations:** 1 Institut de Chimie de Nice, Université Côte d’Azur, CNRS, UMR 7272, 06108 Nice, France; Adrien.Garcia@unice.fr (A.D.G.); Cornelia.Meinert@unice.fr (C.M.); Haruna.Sugahara@unice.fr (H.S.); 2 Japan Aerospace Exploration Agency–Institute of Space and Astronautical Science, 3-1-1 Yoshinodai, Chuo Sagamihara, Kanagawa 252-5210, Japan; 3 ISA, Department of Physics and Astronomy, Aarhus University, 8000 Aarhus C, Denmark; nykj@phys.au.dk (N.C.J.); vronning@phys.au.dk (S.V.H.)

**Keywords:** amino acids, sugars, chirality, comets, meteorites, interstellar

## Abstract

The biomolecular homochirality in living organisms has been investigated for decades, but its origin remains poorly understood. It has been shown that circular polarized light (CPL) and other energy sources are capable of inducing small enantiomeric excesses (*ee*s) in some primary biomolecules, such as amino acids or sugars. Since the first findings of amino acids in carbonaceous meteorites, a scenario in which essential chiral biomolecules originate in space and are delivered by celestial bodies has arisen. Numerous studies have thus focused on their detection, identification, and enantiomeric excess calculations in extraterrestrial matrices. In this review we summarize the discoveries in amino acids, sugars, and organophosphorus compounds in meteorites, comets, and laboratory-simulated interstellar ices. Based on available analytical data, we also discuss their interactions with CPL in the ultraviolet (UV) and vacuum ultraviolet (VUV) regions, their abiotic chiral or achiral synthesis, and their enantiomeric distribution. Without doubt, further laboratory investigations and upcoming space missions are required to shed more light on our potential extraterrestrial molecular origins.

## 1. Introduction

### 1.1. The Asymmetry of Life

Life’s origin remains one of the most enigmatic and fascinating phenomena, requiring a thoughtful scientific explanation. Throughout history, people all over the world have been interested in the beginnings of biological evolution, and many theories, based on science or not, have been elaborated. From the molecular point of view, it is fundamental to discuss the inherent asymmetry of important biological molecules whose origins are still unknown [1]. Indeed, left-handed (l) amino acids make up proteins, whereas the biopolymers RNA, DNA, or even amylum and cellulose are made of right-handed (d) sugars [2]. However, abiotic synthesis usually leads to racemic compositions of asymmetric molecules. This statement leads to some fundamental questions we need to answer: How and why did life choose one handedness over the other and how and where did chiral molecules form?

Several investigations have focused on processes capable of inducing molecular symmetry breaking. As examples, we note symmetry-breaking by crystallization [3], the enantioselective adsorption of amino acids on mineral surfaces [4], the enantioselective synthesis on chiral quartz [5], or the parity violation in the weak interaction [6]. However, circularly polarized light (CPL) appears to be the most promising candidate to induce enantiomeric excesses (*ee*s) in a chiral compound under astrophysical conditions. Consequently, this review will highlight the latest progress in the asymmetric formation of chiral biomolecules by means of CPL and their detection in simulated, as well as authentic, extraterrestrial matter.

### 1.2. Circularly Polarized Light (CPL): A Chiral Bias

CPL is an electromagnetic wave which can be viewed as being composed of two perpendicular linearly polarized electromagnetic field vectors of equal amplitude but offset from each other by a 1/4 wavelength. Thus, CPL possesses chirality, as it can be right-handed (*r*-CPL) or left-handed (*l*-CPL) according to the direction of rotation [7].

Measurements of circular dichroism (CD) spectra of chiral molecules reflect the difference in interaction, with *r*-CPL and *l*-CPL induced by their enantiomeric configuration [8]. As a matter of fact, CD represents the difference in the molar extinction coefficient (or more simply, in absorption) at a given wavelength between both helicities of CPL by an enantiopure compound:

Δ*ε* = *ε*_(*l*-CPL)_ − *ε*_(*r*-CPL)_

This differential absorption between the two helicities of CPL results in unequal photolysis rates of two enantiomers when irradiated with either left- or right-handed CPL. As a consequence, one enantiomer is photo-destroyed more rapidly than the other [9]. Thus, the chirality of light can induce an enantioselective destruction in a racemic mixture and create an enantiomeric excess of the less absorbing enantiomer.

In the course of the creation of *ee*s in relevant biological molecules, CD spectra of several compounds such as amino acids, amines, sugars, nucleosides, and nucleotides were reliably recorded first in the visible and the ultraviolet (UV) wavelength regions, and later in the vacuum ultraviolet (VUV) due to the development of synchrotron radiation CD spectroscopy [10,11,12,13,14,15,16,17]. The interest in recording CD spectra in the VUV range was to measure the potentially strong absorption bands of molecules below 200 nm, especially for amino acids. These measurements required the preparation of amino acids as amorphous thin solid films to avoid the strong VUV absorption of commonly used solvents. Moreover, the isotropic solid form of chiral biomolecules is proposed to better reflect their natural environment in interstellar ices [18,19,20]. Thanks to this progress, CD spectra of meteoritic α-H amino acids and α-methyl amino acids have been recorded [17,18,19,20].

Another important parameter related to optical activity is the anisotropy factor *g*. It is useful for a quantitative description of the chemical kinetics of enantioselective photoreactions, as it is directly linked to the inequality of two enantiomers taking into account the total molar extinction coefficient [21]. It is expressed as the difference in molar extinction coefficient between *r*- and *l*-CPL (Δ*ε*) divided by the global molar extinction coefficient (*ε*):

$$g=\frac{\varepsilon_{\left(l-\mathrm{CPL}\right)}-\varepsilon_{\left(r-\mathrm{CPL}\right)}}{\varepsilon }=\frac{\Delta \varepsilon }{\varepsilon }=\frac{\Delta \varepsilon }{(\varepsilon_{\left(l-\mathrm{CPL}\right)}+\varepsilon_{\left(r-\mathrm{CPL}\right)})/2}.$$

The anisotropy factor *g* with values ranging from 0 < *g* < 2 can also be expressed with the kinetic constants of each enantiomer. d and l, as kinetic constants, are proportional to molar extinction coefficients [22]:

$$g=2\frac{\varepsilon_{D}-\varepsilon_{L}}{\varepsilon_{D}+\varepsilon_{L}}=2\frac{k_{D}-k_{L}}{k_{D}+k_{L}}.$$

The induced *ee* can be predicted from the anisotropy factor *g* and the extent of the photolysis rate ξ as an upper bound of *ee* in the lower limit of *g* by the following inequation [22]:

$$ee\geq \left(1-{\left(1-\xi \right)}^{\frac{g}{2}}\right)\times 100.$$

At the present time, anisotropy spectra of several amino acids in their amorphous solid state and in the VUV–UV region are known [22,23]. The recorded proteinaceous amino acids—alanine (Ala), valine (Val), leucine (Leu), serine (Ser), proline (Pro)—generally exhibit minima and maxima in the same wavelength regions, giving rise to the possibility of inducing the same sign of *ee* in all proteinaceous amino acids at a given wavelength and helicity. For instance, *r*-CPL at 185 nm induces an l-*ee* in all of these amino acids.

Enantioselective photolysis was first carried out experimentally by Kuhn and Braun through irradiation of a racemic mixture of α-bromopropanoic acid ethyl ester in solution [24]. Almost 50 years later, the first enantioselective photolysis of a racemic amino acid was conducted in solution, leading to −2.5% *ee* for *l*-CPL and 1.98% *ee* for *r*-CPL, both at 212.8 nm, with a decomposition of 59% and 75%, respectively [25].

**Figure 1:**
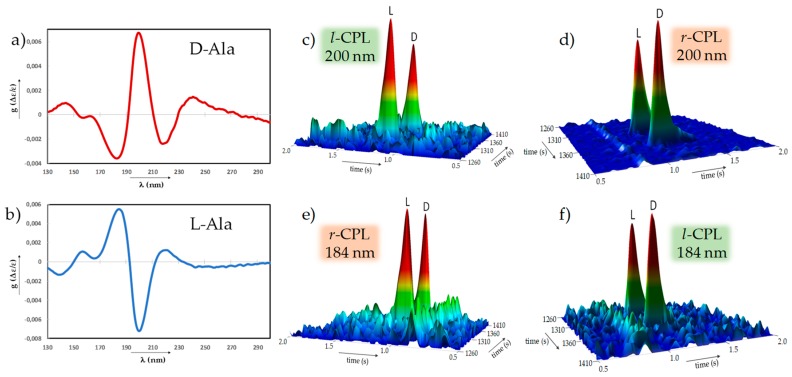
Energy- and polarization-dependent CPL-induced enantiomeric excesses. (**a**, **b**) Anisotropy spectra in the vacuum UV and UV spectral range of enantiopure d-alanine (**a**) and l-alanine (**b**) in the solid state. (**c**–**f**) Close-up view of the multidimensional enantioselective gas chromatographic analysis of ^13^C-alanine enantiomers, after irradiation with CPL differing in helicity and energy. Amorphous racemic ^13^C-alanine was irradiated at 200 nm (6.19 eV), corresponding to the anisotropic maximum (**a**,**b**), with *l*-CPL resulting in a l-*ee* (**c**), and *r*-CPL resulting in the opposite *ee* value (**d**). Irradiation of amorphous racemic ^13^C-alanine at a wavelength of 184 nm (6.74 eV) with *r*- and *l*-CPL also induced opposite *ee*s but they were flipped with regard to the wavelength of irradiation (**e**,**f**). CPL: circularly polarized light; UV: ultraviolet; *ee*: enantiomeric excess.

Later, enantioselective photolysis in the VUV became possible by using synchrotron radiation, along with solid films of amino acids. Racemic films of Leu irradiated with *r*- and *l*-CPL at 182 nm gave opposite *ee*s (2.6% and −0.88%, respectively), in accordance with the CD spectra of Leu where the π*→π_1_ electronic transition is at 183 nm [26]. Conversely, irradiation at 170 nm did not give opposite and significant *ee*, as no CD band is observed at this wavelength. The highest reported induced *ee* so far (5.2%) for racemic Leu was achieved by irradiation with *r*-CPL at 187 nm and a decomposition rate of 99.23% [27]. Unfortunately, the determination of the *ee* after *l*-CPL irradiation was not feasible due to an insufficient quantity of remaining amino acid. However, the expected mirror effect between *r*- and *l*-CPL has been observed [28,29]; to ensure no experimental bias due to contamination, isotropic films of ^13^C labelled racemic Ala were prepared and combined with improved analyses through the use of enantioselective multidimensional gas chromatography coupled to a time-of-flight mass spectrometer (GC × GC-TOFMS). The sample was irradiated with *r*- and *l*-CPL at 184 nm and 200 nm, corresponding to the n→π* transition in the carboxylate anion along with the n(COO^−^)→σ*(N-H) transition and to the π→π* transition, respectively. The absolute values of the *ee*s obtained were almost identical but opposite for both wavelengths: 4.19% for *l*-CPL and −4.22% for *r*-CPL at 200 nm, and 3.15% for *r*-CPL and −3.12% for *l*-CPL at 184 nm, all with very similar extent of reaction rates ξ ranging from 99.93% to 99.97% (Figure 1).

Recently, enantioselective photoreactions were successfully extended to the gas phase by photo-electron circular dichroism (PECD) [30]. Tia et al. irradiated α-Ala with CPL at lyman-α (121.6 nm) and reached an asymmetric flux of gaseous Ala cations, that is to say, an *ee* of 4%. The authors proposed an astrophysical scenario in which such a flux could be neutralized and incorporated into comets and asteroids to further seed the Earth.

### 1.3. The Astrophysical Scenario

The discovery of *ee*s in meteoritic amino acids prompted the search for stellar CPL sources in the universe. Several sources, such as synchrotron radiation from magnetic neutron stars, high field magnetic white dwarfs, and polars (magnetic white dwarf binaries) were considered but rapidly dismissed [31,32,33]. The most plausible CPL source appeared to be the reflection nebulae in star-forming regions [33].

In the near infrared, CPL of up to 17% was detected within the high mass star-forming regions of the Orion Molecular Cloud (OMC-1) [34]. Fukue et al. measured the CPL area around the Orion BN/KL region to be much larger than the size of our solar system (approximatively 0.4 pc) [35]. Higher CPL (22%) with a larger spatial distribution (0.65 pc) has been detected in the massive star-forming region NGC 6334-V [36].

CPL in the interstellar medium could originate from several phenomena: (1) multiple scattering of linearly polarized light from spherical grains, (2) scattering from magnetically-aligned non-spherical grains, and (3) dichroic extinction of scattered linearly polarized light [36]. So far, CPL in the VUV or even UV region has never been observed in star-forming regions, due to dust shielding. Yet, it has been proved in calculations by Bailey et al. that the two first mechanisms plausibly responsible for circular polarization of light could be extended to the UV region [34].

Our solar system was probably formed within a massive star-forming region [37,38], where the presence of CPL is certified [33,34,35]. As such CPL regions are much larger than the size of the solar system, organic molecules could have been asymmetrically synthesized by the interaction with CPL. Then, these molecules may have been synthesized on the surface of small dust grains within the solar nebula and later embedded in small celestial bodies such as parent meteorites and comets, and eventually delivered to the early Earth when the solar system was formed. Alternatively, molecule formation may have taken place after accretion of the small dust grains into larger objects. This hypothesis is—due to the limited penetration depth of the CPL—thought to involve non-photochemical mechanisms such as hydrolysis and thermolysis.

In the following chapters we summarize the findings on three important classes of biomolecules that share an astrophysical origin and might have impacted the prebiotic pathways on the early Earth: amino acids (Section 2) and sugars (Section 3). Each section discusses their discovery in: meteorites (Section 2.1 and Section 3.1), comets (Section 2.2 and Section 3.2), and simulated interstellar ices (Section 2.3 and Section 3.3).

## 2. Amino Acids

### 2.1. Amino Acids in Meteorites

Meteorites are extraterrestrial bodies that generally originate from the fragmentation of larger objects such as asteroids. They have been subject to many studies since the 19th century for their contents of organic compounds and because they are the most accessible type of extraterrestrial matter. According to their composition and texture, stony meteorites can be separated in two classes; chondrites and achondrites [39]. Chondrites are recognized by the presence of small morphological particularities known as chondrules. They are relatively unaltered and thus a powerful source of information on the early solar system. Contrariwise, non-chondritic meteorites do not possess chondrules because they underwent diverse processes that denatured them [40]. The subclass of carbonaceous chondrites appears to be the most interesting in the field of the origins of life, not only due to their high carbon content, but also because most of this carbon dwells as insoluble organic material [41].

The first studies on carbonaceous chondrites concerned the Alais, Kaba, and Orgueil meteorites and highlighted the presence of organic compounds [42,43,44]. Despite some likely contaminations, these preliminary surveys triggered a keen interest in life’s origin on extraterrestrial bodies and gave birth to the initial postulation that carbonaceous chondrites contain polymeric organic materials [45].

The fall of the Murchison meteorite in 1969 gave a strong boost to the search for extraterrestrial organic compounds. Right after the fall of this CM2 (Mighei type) carbonaceous chondrite, Kvenvolden et al. discovered, after derivatization and analyzes by GC-MS, seven amino acids (glycine (Gly), Ala, Val, Pro, glutamic acid (Glu), 2-methylalanine, and sarcosine) [46]. The high isotopic ^13^C/^12^C ratio (compared with that found on Earth) combined with the fact that both enantiomers of all five chiral amino acids were present in almost equal amounts strongly suggested an extraterrestrial origin and a very low contamination rate. On the other hand, the racemic composition of chiral amino acids and the presence of sarcosine and 2-methylalanine implied their abiotic synthesis as they are less abundant on Earth. These findings were corroborated in 1971 by the discovery of new amino acids in racemic mixtures in the Murchison meteorite (aspartic acid (Asp) and seven non-proteinogenic amino acids) and the Murray meteorite (17 amino acids, 11 of which were non-proteinogenic) [47,48]. In this set of amino acids, the presence of both enantiomers of *iso*-valine (iVal) excludes any racemization process by a common mechanism since iVal does not possess a hydrogen atom on the α-carbon atom. Therefore, iVal and the other amino acids were supposed to be synthesized as racemates, under abiotic conditions [49].

The quest for the amino acid composition of the Murchison meteorite has continued, such that in the 1980s Cronin et al. identified six of the seven six-carbon acyclic α-amino acids (chain isomers of Leu) [50], all five-carbon acyclic β, γ, and δ-amino acids (position isomers of valine) [51], and all seven-carbon acyclic α-amino acids [52], by ion exchange chromatography, gas chromatography-mass spectrometry (GC-MS), and reverse phase chromatography. The authors were able to make some important observations: (1) the quantities of amino acids decrease with the increase of the carbon number; (2) α-amino acids are the most abundant amino-acids followed by γ and then β-amino acids; and (3) branched carbon chain amino acids are more abundant than linear chain ones [50,51,52].

More recently, Meierhenrich et al. identified diamino acids in the Murchison meteorite, by GC-MS analysis of the *N*,*N*-diethoxycarbonyl ethyl ester derivates. This is a class of molecules relevant for the appearance of life since they are thought to be the starting point for the synthesis of peptide nucleic acid (PNA), a possible ancestor of RNA and DNA [53,54]. More than a decade later, 10 new amino acids were identified in the Murchison meteorite, nine of which are hydroxy amino acids, a new family in meteorites [55]. Nowadays, 96 amino acids exhibiting a remarkable structural diversity with α, δ, γ, and δ isomers of C_2_ to C_9_ simple amino acids, diamino acids, and dicarboxylic acids have been identified in this meteorite.

The various types of meteorites differ in their composition of amino acids. For instance, it is accepted that the least aqueously altered chondrites possess the highest amino acid concentration (249 and 180 ppm for, respectively, the Graves Nunataks (GRA) 95229 and the Elephant Moraine (EET) 92042), whereas the CR1 (Renazzo type) Grosvenor Mountains (GRO) 95577, the most altered CR chondrite, contains the lowest amino acid concentration [56]. Moreover, β-alanine (β-Ala) is the most abundant amino-acid in CI (Ivuna type) chondrites, and CV (Vigarano type)/CO (Ornans type) chondrites are enriched in straight-chain amine terminal (n-ω-amino) amino acids, whilst there is a predominance of α-amino acids in the Murchison meteorite [57,58,59]. These differences in distribution of amino acids can be explained by aqueous alterations on the meteorites or their parent bodies, but also by the region of formation of the parent bodies which affected the precursor compositions and syntheses of amino acids [57,60].

The first *ee*s reported in meteorites concerned five proteinogenic amino acids extracted from the Murchison meteorite by Engel and Nagy in 1982, but shortly afterwards these discoveries were shown to be the result of bacterial contamination [61,62]. Later, small l-*ee*s were discovered by Pizzarello and Cronin in six rare, or even missing on Earth, α-methyl amino acids of the Murchison (2.8–9.2% (±0.3–1.3%)) and Murray (1.0–6.0% (±0.3–0.7%)) meteorites [63,64,65]. Conversely, they observed no *ee* for the four α-hydrogen-α-amino alkanoic acids. It has been proposed that α-hydrogen amino acids could have undergone racemization much faster than α-methylated amino acids because of their labile hydrogen atom at the chiral alpha carbon atom. The methyl group in α-methyl amino acids, in contrast, prevents racemization. This class of amino acids can therefore maintain any initially induced *ee*. A plausible cause of this asymmetry could be the irradiation by UV-CPL in space. The detailed investigation of the 5-carbon amino acids (valine isomers) in CM, CR, and CI meteorites led Glavin and Dworkin to discover the largest l-*ee*s reported so far [66]. Indeed, they detected l-*ee* of 18.5% (±2.6%) in the CM2 Murchison meteorite and 15.2% (±4.0%) in the CI Orgueil meteorite for the α-methyl amino acid iVal. The absence of l-iVal enrichment in the unaltered Antarctic CR meteorites EET 92042 and QUE 99177 supports the theory that amplification of initially small *ee*s by aqueous alteration on the meteorite parent bodies is responsible for the high l-*ee* values found in certain amino acids. The authors discussed that the delivery of l-enriched amino acids to the early Earth by fragments of asteroids and comets may have influenced Earth’s molecular reservoir and its evolution up to life.

Low concentrations and the high complexity of meteoritic amino acids act as a serious drawback for their enantioselective analysis. Once extracted, the organic material must be hydrolyzed under acidic conditions. At this stage, the resulting amino acids are fractionated by ion exchange chromatography and then derivatized to be baseline separated, detected and quantified on GC or LC systems. Nowadays, comprehensive multidimensional GC or LC systems are taking precedence over one-dimensional techniques because they clearly outperform the limitations of 1D-GC and LC in terms of selectivity and resolution [67,68].

A multidimensional gas chromatography method for the separation and quantification of non-racemic samples of amino acids has been developed and extended to the detection and quantification of non-proteinogenic amino acids in extraterrestrial samples [69,70]. Prior to the analysis of an acid-hydrolyzed extract of the Murchison meteorite by enantioselective two-dimensional gas chromatography coupled to a time-of-flight mass spectrometer (GC × GC-TOFMS), the amino acids were transformed into their *N*(*O*,*S*)*-*ethoxycarbonylheptafluorobutyl-corresponding esters. With this method, the l-*ee* values of 19 amino acids ranging from C_3_ to C_9_ were determined to be equal to 0 or positive up to 26.33% (±0.76%) for Leu, with one exception being *allo*-isoleucine (*allo*-iLeu) (−9.55% (±0.70%)). In accordance with previous studies, the highest *ee*s were found for α-methyl amino acids in the class of non-proteinogenic amino acids [71].

Amino acid enantiomers can also be separated by two-dimensional chiral HPLC systems as proven by Hamase et al. in 2014 [72]. They validated their method with the analysis of extraterrestrial amino acids from the CM2 meteorite Yamato 791191. The amino acids were derivatized with 4-fluoro-7-nitro-2,1,3-benzoxadiazole (NBD-F) and then injected in the 2D-HPLC system. The first dimension consisted of an achiral reverse-phase separation whose outgoing fractions were automatically submitted to the second dimension to be separated by Pirkle-type chiral columns. Finally, they were detected and quantified by fluorescence as NBD-F derivatives [73]. Surprisingly, the eight amino acids analyzed showed racemic ratios, perhaps indicating the presence of racemic amino acids in the region of formation of the parent body of Yamato 791191.

The detailed synthetic formation pathway of meteoritic amino acids is still unknown. One of the most likely scenarios involves the Strecker synthesis from aldehydes, ketones, amines and hydrogen cyanide (HCN) [66]. The discovery of several ketones and aldehydes in meteorites is then an important piece of information [74,75,76,77,78,79], as it strengthens the hypothesis that meteoritic amino acids result from a Strecker-type reaction. However, this pathway encounters some drawbacks—discussed in Section 2.3—that need clarification.

Despite the huge scientific advances in the past decades, meteoritic amino acids and their asymmetric configuration remain a source of unanswered questions. In the next years, the technological developments in space exploration as well as in powerful separation and analysis techniques will help corroborate or refute theories that have emerged from recent results.

### 2.2. Amino Acids in Comets

Comets are small bodies formed 4.6 billion years ago when the condensation of the solar nebula created our solar system [80,81]. Essentially made up of ice and dust, they are considered to be the most pristine and unaltered bodies, remnants of the early solar system [82]. They are therefore of great interest for learning more about the early stages of the solar system and for deciphering the events that led to the emergence of life [83]. Indeed, comets are seriously suspected of being responsible for the delivery of organic molecules (precursors of biomolecules) on early Earth, and even more so since the first discoveries of relevant biomolecules for the appearance of life in simulated interstellar ices (further discussed in Section 2.3 and Section 3.3.

Unlike meteorites, comets are very inaccessible objects and studying them is much more complicated than for meteorites. In 1986, Schloerb et al. reported the first observation of HCN from comet Halley by spectroscopy [84]. The Giotto mission targeting this comet allowed the discovery of organic matter consisting of an aggregation of acetylene (C_2_H_2_), formaldehyde (CH_2_O), and HCN in the cometary dust and coma [85]. The presence of HCN in cometary matter is very important as it can react with aldehydes or ketones and amines to yield α-amino acids by Strecker synthesis [66]. Radio spectroscopy studies failed to observe amino acids in comets. In 2004, Crovisier et al. reported the observation of several molecules, primarily from C/1996 B2 (Hyakutake) and C/1995 O1 (Hale-Bopp) but were incapable of detecting Gly, the most abundant amino acid in CI and CM chondrites [86].

The National Aeronautics and Space Administration (NASA) and the European Space Agency (ESA) developed two major missions on comets; Stardust and Rosetta respectively. The first mission returned, for the first time, a cometary dust sample from the coma of comet 81P/Wild 2 back to Earth. The analysis of the sample showed the presence of Gly, l-Ala, β-Ala, γ-amino-n-butyric acid (GABA), and ε-amino-n-caproic acid (EACA), but after determination of the d/l and isotopic ratios only Gly was confirmed to be extraterrestrial.

The second mission aimed to survey the comet 67P/Churyumov-Gerasimenko and planned the first landing on the nucleus of a comet [87]. The lander Philae was supposed to land on the “Agilkia” site, but because of the non-nominal operation of the Active Descent System (ADS) and the two anchoring harpoons, it touched down and bounced several times on the cometary surface to reach its final landing site “Abydos” [88]. As Philae was resting on its side, the Sample Drilling and Distribution (SD^2^) instrument could not collect any samples of the cometary surface for the scheduled analysis with the COmetary SAmpling and Composition (COSAC) enantioselective gas chromatograph, which was coupled to a time-of-flight mass spectrometer (TOF-MS). The COSAC instrument was, however, able to operate without the SD² system and without chromatographic separations in a so-called “sniffing mode” [87]. This was done before Philae reached its final destination, immediately after the first touchdown, as well as while approaching comet 67P to record several background spectra. The recorded mass spectrum after “landing” exhibited many more peaks than the previously taken background spectra and their intensities were much higher. This was due to the cometary matter excavated during the first collision with the surface of the comet that had entered the system and been analyzed. The authors have searched for the best match to this spectrum by superimposing National Institute of Standards and Technology (NIST) mass spectra of potential cometary molecules. The best fit was finally a mixture of 16 molecules including HCN, ketones, aldehydes, and amines which are key molecules for the prebiotic synthesis of amino acids [89].

In addition, the mass spectrometer Rosetta Orbiter Spectrometer for Ion and Neutral Analysis (ROSINA) on board the Rosetta mother spacecraft detected Gly (75 Da), the simplest amino acid, in the coma of 67P/Churyumov-Gerasimenko, confirming the Stardust results [90].

Even though Gly is so far the only amino acid found in comets, the Rosetta results proved that a cometary nucleus contains all of the precursors of amino acids needed. Future space explorations combined with the study of laboratory simulated interstellar ices, a topic discussed in the next subchapter of this review, will hopefully extend the knowledge about comets and their molecular inventory.

### 2.3. Amino Acids in Simulated Interstellar Ices

Interstellar clouds are composed of small size particles called interstellar dust grains dwelling in a gaseous mixture of molecules and atoms [91]. Because of the very low temperature, this gaseous mixture—containing carbon, nitrogen and oxygen-based molecules—condenses on the surface of the dust grains to form interstellar ices or the so-called icy mantle. UV irradiation, as well as cosmic rays, are believed to trigger diverse reactions of the smaller condensed species, forming more complex molecules. Knowing that stars and planets, along with asteroids or comets, were born in these regions, interstellar chemistry, particularly in the ices, might be one of the most relevant sources of organic molecules and could provide tremendous information on the composition of the early solar system [92].

**Figure 2:**
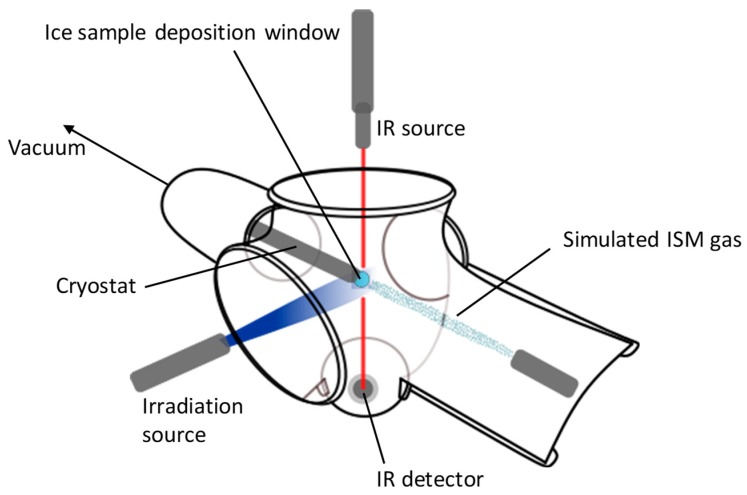
Interstellar ice simulation chamber. Interstellar-like gas molecules are deposited on the cooled ice sample deposition window and simultaneously irradiated by an energy source. IR (Infrared) analyses are performed to control both composition and thickness of the ice.

Since interstellar ices are difficult to characterize by radio astronomy alone, several studies focus on replicating and simulating the interstellar conditions and environments in the laboratory. To be consistent, these experiments are, in almost all cases, performed at very low temperature (10–80 K) and under ultra-high vacuum [92,93]. Typical interstellar gases—which were found to be H_2_O, CH_3_OH, NH_3_, CO, and CO_2_ by infrared (IR) observations [94]—are condensed on a cold substrate and simultaneously irradiated with different kinds of radiation at different energies to trigger photochemical processes (Figure 2) [95].

In 2002, Bernstein et al. condensed a mixture of H_2_O:CH_3_OH:NH_3_:HCN (20:2:1:1) on a nickel substrate under interstellar conditions (15 K, 10^−8^ torr and UV radiation) [96]. Gly, Ala, and Ser were identified in the resulting organic residue after hydrolysis. Any potential contamination was excluded as the GC-MS analysis showed fully ^13^C labelled amino acids and HPLC analysis resolved the enantiomeric distribution by transforming enantiomers into diastereomers by reaction with a chiral label. Ala and Ser were found to be in racemic proportions, confirming that the experiment was free from contamination.

Simultaneously, another team performed a similar experiment with a mixture of H_2_O:CH_3_OH:NH_3_:CO:CO_2_ (2:1:1:1:1) irradiated with Lyman-α photons and detected with enantioselective GC-MS a total of 16 amino acids, among which six were proteinogenic (Gly, Ala, Val, Pro, Ser and Asp) and four are diamino acids [97]. Once again, isotopic ^13^C labelling proved the results to be reliable.

Glycine, the simplest and achiral amino acid, was the most abundant in both studies. Both teams also noted the contribution of the hydrolysis process on the increased abundances of amino acids. These observations gave birth to the postulation that the product of such experiments is peptidic or otherwise bound molecules. It is also important to note that some of these identified amino acids were found in earlier studies in meteorites [46,53,98].

Nuevo et al. investigated the effect of five parameters—the irradiation time, the temperature, the ice composition, the photon dose per molecule and the deposition substrate—on the final amino acid composition of the interstellar ice analogue [99]. They concluded that the amino acid quantities are affected by the composition of the ice mixture and the photon dose per molecule affects the final distribution. The three other factors were found not to be significant.

Different energy sources have been studied for the interstellar-like synthesis of amino acids such as vacuum UV irradiation [100,101,102,103], γ-rays [101,102], X-rays [104], high-energy particles [105], protons [106], and electrons [107], with some experiments carried out at room temperature [108].

Meteoritic amino acids were thought to come from the Strecker synthesis, a reaction involving HCN, NH_3_, and an aldehyde in aqueous medium [66]. However, this reaction can only explain the formation of α-amino acids, and discrepancies like the inconsistent deuterium enrichments between hydroxy acids and amino acids due to deuterium exchange with deuterium-enriched meteoritic water [109] are found. Thus, alternative pathways were needed and simulated interstellar ices were studied for this purpose. In 2002, Woon explored radical chemistry to explain the formation of Gly, Ala, and Ser [110]. He concluded that the carbon of the carboxylic group originates either from carbon monoxide (CO) or methanol (CH_3_OH), whereas the CH_2_NH_2_ moiety originates from hydrogen cyanide (HCN). The combination of the two fragments directly yields Gly. The syntheses of Ala and Ser require an additional step: ejection of one hydrogen from the α-carbon and the combination with CH_3_ or CH_2_OH, both fragments being products of methanol photolysis.

After this, Elsila et al. explored the isotopic (^13^C and ^15^N) distribution of their produced Gly and Ser to explore the contributions of each simple starting molecule [111]. They found that none or one carbon atom stems from CH_3_OH and one or two from HCN, as well as the nitrogen atom in most cases. The carbonyl group is mainly originating from HCN, the central carbon can be provided from HCN or CH_3_OH with almost the same probability. Ser follows a similar pattern, with its nitrogen atom, acid carbon and central carbon from HCN, whereas CH_3_OH mostly supplies the side chain carbon atoms. NH_3_ was found not to be essential for the production of Ser and Gly, but yields were very low without it, making the author suggest a catalytic role. In conclusion, they excluded the Strecker synthesis as it plays a negligible role in Gly synthesis and supported instead a radical–radical mechanism including nitriles.

In a more recent study, Oba et al. investigated the question by studying the D/H isotopic ratio resulting from the UV/VUV photon photolysis of a mixture containing singly deuterated methanol CH_2_DOH along with H_2_O, CO, and NH_3_ (2:5:2:2) [112]. They successfully synthesized five amino acids which were divided into two groups in their study: those in which the D/H ratios remained unchanged (Gly, sarcosine, and Ser), and those in which the D/H ratios significantly decreased (α-Ala and β-Ala) after hydrolysis. This made them conclude that interstellar amino acids probably form by two different photo-induced mechanisms.

Nuevo et al. reported the first investigations on the amino acid *ee*s in interstellar ice analogues [113,114]. After irradiation of an ice mixture of H_2_O:^13^CH_3_OH:NH_3_ (1:1:1) by *r*-CPL or *l*-CPL at 167 nm, they measured very small *ee*s of up to 1% for Ala and 2,3-diaminopropanoic acid (DAP).

Using proton irradiation on a gaseous mixture of CO, NH_3_, and H_2_O followed by VUV-CPL irradiation on the resulting macromolecular compounds, Takano’s team succeeded in synthetizing 11 amino acids, among which Ala was found in opposite small *ee*s (less than 1%) for *r*-CPL and *l*-CPL and as racemates without CPL irradiations [115].

**Figure 3:**
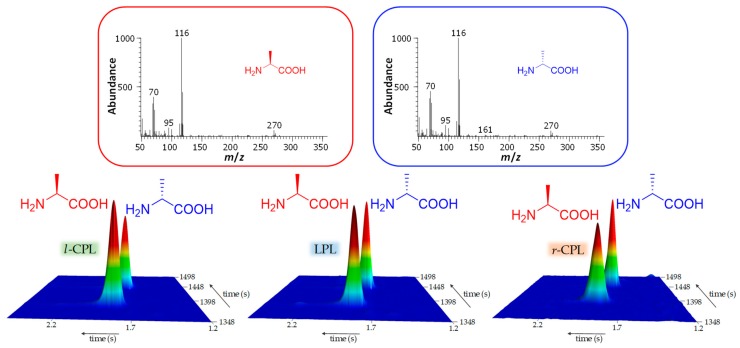
Multidimensional gas chromatograms and mass spectra of ^13^C-alanine enantiomers for the three polarization regimes. From left to right, amino acids l-alanine (drawn in red) and d-alanine (drawn in blue) produced by the 6.64 eV UV photo-irradiation with left-handed circularly polarized light (*l*-CPL), linearly polarized light (LPL), and right-handed circularly polarized light (*r*-CPL). The alanine produced by irradiation with *r*- and *l*-CPL shows opposite enantiomeric excesses, whereas the irradiation with LPL shows no enantiomeric excess as expected. Mass spectra for both enantiomers, given at the top of the figure, are identical.

Later on, de Marcellus et al. used enantioselective comprehensive multidimensional gas chromatography coupled with a time-of-flight mass spectrometer (GC × GC-TOFMS) to resolve the enantiomeric distribution in an UV-CPL irradiated ice mixture of H_2_O:^13^CH_3_OH:NH_3_ (2:1:1) [116]. Enantiomeric excesses of up to 1.34% were measured for ^13^C-Ala. Moreover, they found a correlation between, on one hand the sign of the induced *ee* and the helicity of CPL, and on the other hand the absolute values of the induced *ee* and the number of photons per molecule (Figure 3).

It should be noted that the wavelength of the UV-CPL was more pertinent and was calculated to match the n→π* transition. These results were confirmed shortly after when *ee*s between –0.20% and −2.54% with *l*-CPL irradiation were measured for five amino acids using enantioselective GC × GC-TOFMS [117]. They were obtained by irradiating interstellar ice analogues with CPL at different wavelengths (121.6 nm and 187.9 nm). Same signs were induced on the five amino acids for given wavelength and helicity of the CPL and the two wavelengths induced opposite signs as suggested by the anisotropy spectra [28,118].

Through all these experiments, photochemical processes have been proven to be serious candidates for triggering the synthesis of amino acids in the interstellar medium as well as CPL for the appearance of symmetry breaking. Thus, interstellar clouds might not only be the birth place of one of the most relevant classes of molecules in life, but also the site where their molecular symmetry breaking took place, resulting in the appearance of slight *ee*s. Their delivery on Earth and especially their chiral amplification remains somewhat unclear—even if some hypotheses are plausible—and would need additional studies. The scientific community also looked for other types of biomolecules in simulated interstellar ices, as discussed in Section 3.

## 3. Chiral Sugars

### 3.1. Chiral Sugars in Meteorites

Meteoritic sugar-related literature is much scarcer than for amino acids. However, they have been studied for more than 60 years, before the Murchison meteorite’s fall. Degens and Bajor detected sugars up to 20 ppm in the non-chondritic Bruderheim meteorite, and 70 ppm in the Murray meteorite [119]. In a subsequent study of a set of 14 meteorite samples, sugars were detected and quantified in the carbonaceous chondrites by thin-layer chromatography [120]. These sugars were glucose, mannose, xylose, and arabinose, and their total concentration varied from 5 to 26 ppm. Optical experiments were conducted but no activity was recorded, suggesting an abiotic synthesis pathway. The chromatographic methods used at that time, however, were limited and the experiments likely subject to contamination [45].

After these publications, meteoritic sugars were neglected for a long time. In 2001, Cooper et al. identified one achiral sugar and a large variety of chiral polyols including sugar alcohols, sugar acids, dicarboxylic sugar acids, and deoxy sugar acids with three to six carbon atoms in the Murray and Murchison meteorites by GC-MS [121]. The detection in sufficient amount of rare and non-biological sugar derivatives, such as 2-hydroxymethylglycerol and 2-hydroxymethylglyceric acid, supported the abiotic origins of these compounds. As for meteoritic amino acids, sugar derivatives followed a global decrease in abundance with increasing number of carbon atoms. Certainly, due to the basic conditions used before derivatization, no sugars except the glyceraldehyde isomer dihydroxyacetone were found. Although the two-carbon polyol ethylene glycol was detected, glycolaldehyde or its oxidation product glycolic acid were not seen.

**Figure 4:**
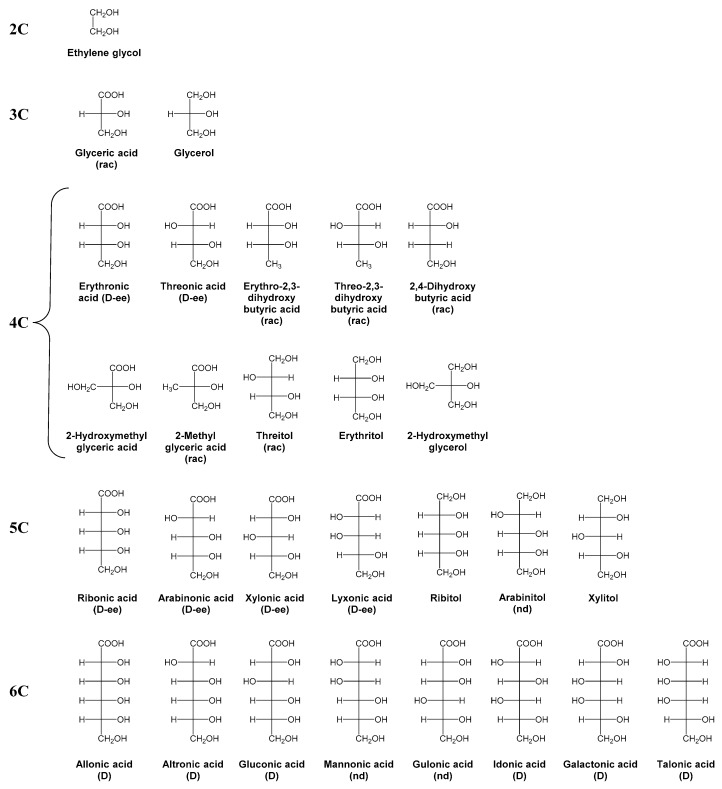
Structures and names of identified sugar derivatives in Cooper and Rios [122]. “rac” indicates sugar derivatives found in racemic distribution, “d-*ee*” indicates sugar derivatives found with an enantiomeric excess of the d-form, “d” is for sugar derivatives only detected in their d-forms, “nd” means the enantiomeric excess could not be determined, and those with no indication are achiral.

Unlike amino acids, the most displayed enantiomeric form of sugar molecules in biological systems is the d-form. For instance, we could cite the well-known d-ribose and d-2-deoxyribose, constituents of DNA and RNA. This homochirality is of the same utmost interest as the left-handedness in amino acids in the search for the origin of life. The first chiral analyses of meteoritic sugar derivatives were performed very recently by Cooper and Rios using chiral GC-MS [122]. They determined a racemic distribution for the only chiral 3C sugar derivative, glyceric acid, which is consistent with an abiotic origin (Figure 4). In contrary, 4C threonic acid is predominantly present as the d-enantiomer, with enantiomeric excesses between 33% and 55%. This sugar acid is likely not a contaminant since it is less abundant than glyceric acid in nature. In the case of 4C sugar alcohols, threitol, the only chiral one, was found as racemates, unlike 4C sugar acids, indicating potentially different synthesis pathways or different evolution after synthesis. Despite their low abundances, the authors found d-*ee*s up to 82% for the 5C sugar acids. The d-*ee* of arabinonic acid is particularly significant since l-arabinonic acid and l-arabinose are more common than their antipodes. Once again, one can notice that lyxonic acid, a biologically rare sugar acid, also displayed large d-*ee*, bolstering the absence of contamination in the experiments. Due to its low abundance, the d/l distribution of arabinitol was impossible to determine. The same problem was encountered for 6C sugar acids. However, six of them were identified and even though d/l ratio could not be measured, only the d-enantiomers were observed supporting potential d-*ee*s of 6C meteoritic sugars. d-forms of 6C sugar acids are expected to be proportional to the l-form, in larger quantities than for 5C and 4C sugar acids since the overall distribution of sugars in the study followed a general pattern; decreasing abundances and increasing d-*ee*s with increasing carbon number. However, further experiments are essential to confirm this hypothesis.

**Figure 5:**
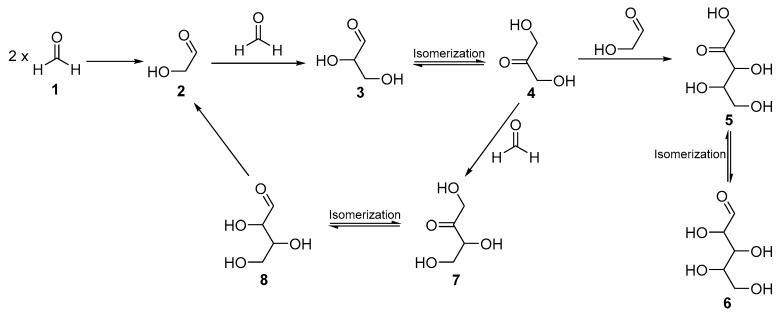
The formose reaction: Formaldehyde (**1)** undergoes self-condensation to form glycolaldehyde (**2**). Glyceraldehyde (**3**) is formed by aldolization between glycolaldehyde (**2**) and formaldehyde (**1**). Isomerization of (**3**) gives dihydroxyacetone (**4**) which reacts with (**2**) to form pentulose (**5**) which forms aldopentose (**6**) by isomerization. Dihydroxyacetone (**4**) can also give ketotetrose (**7**) in equilibrium with aldotetrose (**8**) by aldolic condensation with (**1**). Aldotetrose, by reverse aldol reaction, can regenerate two glycolaldehydes which can react again to form (**3**) or (**5**).

Discovered in 1861, the formose reaction is a plausible abiotic pathway to the formation of sugars and their derivatives [123] (Figure 5). It is known as the polymerization of formaldehyde in basic aqueous conditions leading to glycolaldehyde, glyceraldehyde, ribose, glucose, ketone sugars, and so on. However, the reaction cannot happen without initiators such as an enolizable carbon-containing molecule to trigger and/or catalyze the aldolization [124,125]. Glycolaldehyde, the simplest sugar, is an excellent candidate to initiate the reaction and is found together with formaldehyde in several studies [126,127]. Furthermore, both molecules are found in carbonaceous meteorites, strengthening the hypothesis of a formose reaction within the parent body of meteorites during aqueous alteration phases [77,128].

Basic conditions catalyze the process [125]. Strong bases, which are absent in meteoritic matter, are generally used in the laboratory. Milder alkaline conditions would produce lower yields but are sufficient to trigger the formose reaction. Carbonaceous chondrites commonly contain carbonates which are responsible for the slightly basic pH of their aqueous extracts [129,130]. Parent body aqueous alteration periods, when the formose reaction could have been performed, are calculated to range from 20 °C to 71 °C, while laboratory formose reactions are generally made at higher temperatures (50–100 °C) [131]. The combination of lower temperatures and weaker basic conditions in parent bodies may explain the low quantities of sugars and structurally related sugar molecules.

Separation, detection, quantification, and enantiomeric excess calculations of polyols in their original sugar forms (cyclic or linear) in meteorites are still not reported and should be one of the aims of further studies.

### 3.2. Chiral Sugars in Comets

Thus far, the only sugar that was detected in comets is glycolaldehyde, a compound which could potentially trigger the formose reaction thanks to its enolizable carbon [87,125,132]. It was first discovered by in situ mass spectrometry on comet 67P/Churyumov-Gerasimenko [87], and one year later by radio spectroscopy in the comet C/2014 Q2 (Lovejoy) [133]. Research results on comet samples are very limited due to the constraints for reaching them. However, we know that molecules that are precursors of polyols exist within such bodies. The formaldehyde precursor carbon monoxide (CO) was discovered in 1976 thanks to the UV spectra of comet West [134]. Between 1988 and 1991, formaldehyde itself was detected in comet Halley by means of IR, radio spectroscopy and mass spectrometry [134,135,136].

In 2014, the COSAC instrument on board the Rosetta’s lander Philae led to the identification of 16 molecules on comet 67P/Churyumov-Gerasimenko [87]. In addition to glycolaldehyde, CO was found confirming the previous observations. Moreover, they detected acetone, acetaldehyde and propionaldehyde; three carbonyl compounds able to undergo aldolic condensations leading to sugar formation.

In view of the finding of some precursors, one can cautiously assume that sugars and their derivatives can be present in comets.

### 3.3. Chiral Sugars in Simulated Interstellar Ices

Laboratory interstellar ice chemistry has for a long time only been applied for the synthesis and detection of amino acids, but recently aldehydes and sugars were found in such matrices as well [107,137]. Indeed, 10 aldehydes, including simple aldehydes, dialdehydes, and hydroxyaldehydes, which are sugars by definition, were formed by irradiating mixtures of H_2_O:^13^CH_3_OH:NH_3_ (12:3.5:1) and H_2_O:^13^CH_3_OH (3:1) with photons at Lyman-α. The authors highlighted the important role of ammonia for the synthesis of aldehydes, which are produced in higher amounts in the presence of ammonia in the initial gas mixture. Hydroxy aldehydes were even absent without ammonia in the ice mixture. On the contrary, the presence of ammonia decreased the abundances of dialdehydes.

By bombarding with UV photons a mixture of H_2_O:^13^CH_3_OH:NH_3_ (10:3.5:1) at 78 K and very low pressure, the same team successfully synthesized and detected the RNA monomer building block ribose, along with several related sugars and derivatives of sugars, by means of GC × GC-TOFMS [138]. The ribose discovery within artificial interstellar ices was of great interest as its prebiotic origin remained unknown. Very recently, 2-deoxyribose, the monomeric building block of DNA, along with other deoxysugars and deoxysugar derivatives, were discovered in a very primitive interstellar-like ice sample constituted of H_2_O:^13^CH_3_OH (2:1) irradiated with UV-photons [139].

Chirality of sugars in simulated interstellar ices has not been investigated yet. Supplementary experiments involving chiral photons and enantioselective separation of all sugar stereoisomers would be necessary to hypothesize a possible interstellar origin of the asymmetry in biological sugar molecules. In addition, enantioselective analyses of diverse meteorites, as well as in situ or sample return missions of asteroids and comets, will be indispensable to prove the reliability of artificial interstellar ice experiments.

## 4. Conclusions and Perspectives

As amino acids were the original stimuli for the search for chiral biomolecules in extraterrestrial matter, they have been extensively studied over the years. Their existence in carbonaceous meteorites is widely attested and their formation in interstellar-like environments is proven [140]. More importantly, the solid understanding of the amino acid interactions with CPL and its consequences, along with the discovery of CPL in the universe, enabled the scientific community to evolve a plausible scenario where the pathways to biological homochirality was initiated in space. Indeed, CPL would have induced small *ee*s in amino acids and potentially other biomolecules which were later incorporated in comets and asteroids where they would have undergone chiral amplifications before or after their delivery on Earth. By contrast, knowledge of the extraterrestrial origin of other chiral biomolecules such as those discussed in this manuscript (sugars compounds) is much scarcer but advancing. Their potential symmetry-breaking by CPL needs to be investigated to corroborate the feasibility of the above theory. Furthermore, other important biological chiral compounds such as organophosphorous compounds have started to be investigated and may be formed as well in the interstellar media.

Simultaneously, future and ongoing space missions will make their contributions. The Hayabusa2 space mission aims to return to Earth at least 100 mg of surface samples from the carbonaceous-type asteroid (162173) Ryugu [141,142]. The analysis of the data will hopefully provide information about the origin and history of the early solar system. OSIRIS-Rex will return to Earth in 2023 with at least 60 g of a B-type (rare spectral subclass of the carbonaceous-type) asteroid (101955) Bennu [143]. The mission shares a common goal with Hayabusa2: the understanding of the origin and evolution of the solar system, by studying the evolution of water and organic matter in asteroids and their possible involvement in the emergence of life. A sample-return mission targeting comet 67P/Churyumov-Gerasimenko is currently in development and could be selected by NASA to bring a sample of a few hundred grams back to Earth in 2038. As the conditions and morphology of 67P/Churyumov-Gerasimenko are well known, the probability of success is increased, and the mission, if selected, will likely provide complementary information to the Philae results. Finally, the ExoMars 2020 mission will send a lander, the ExoMars rover, to the surface of Mars [144]. The MOMA (Mars Organic Molecule Analyser) instrument onboard the rover includes several analytical features; one is a GC-MS [145,146]. The main goal of the mission is to detect biosignatures of extinct life and signs of the past habitability of Mars.

Hopefully, the knowledge about our origin will be largely enhanced in the next years thanks to all these future and ongoing works.
